# Supplementary material for: Dietary exposures and risk of anxiety and depression symptoms in the Lothian Birth Cohort 1936: a cohort-level GLAD Project analysis
Source: Wellcome Open Res. 2026 Sep 23;10:613. Originally published 2025 Nov 5. [Version 2] doi: 10.12688/wellcomeopenres.24950.2 (PMC13717163; doi:10.12688/wellcomeopenres.24950.2)
Supplement: Peer review history

# Reviewer report: Reviewer response for version 2

**DOI:** 10.21956/wellcomeopenres.30638.r171789

**Published:** 2026-10-28

**Authors:** Monique Aucoin

**Affiliations:** 1 Canadian College of Naturopathic Medicine, Toronto, Ontario, Canada

This cross-sectional study assessed the relationship between diet and mental health outcomes in the Lothian Birth Cohort 1936. The researchers found associations between a small number of foods and anxiety/depression outcomes.

 While limited significant findings were reported in this work, the researchers provided thoughtful and appropriate reasons that might explain such as the low incidence of depression and the overall poor diet quality within the sample.

 “This relatively low prevalence may also reflect characteristics of the cohort itself, including healthy-participant and survival effects, as well as potential generational differences in the recognition or reporting of psychological symptoms.” – this is true and potentially exacerbated by the fact that mental health data was self-reported in the present study. Consider including a reference, such as:(Overend et al., 2015)

 Strengths of this study include use of harmonizable data to allow for comparison and consideration of co-variates. This research is important because it contributes to the limited evidence in older adult populations.

 Overall, this manuscript is well written and describes a well conducted study. The conclusions presented are appropriate.

Is the work clearly and accurately presented and does it cite the current literature?

Yes

If applicable, is the statistical analysis and its interpretation appropriate?

Yes

Are all the source data underlying the results available to ensure full reproducibility?

Yes

Is the study design appropriate and is the work technically sound?

Yes

Are the conclusions drawn adequately supported by the results?

Yes

Are sufficient details of methods and analysis provided to allow replication by others?

Yes

Reviewer Expertise:

nutritional psychiatry

I confirm that I have read this submission and believe that I have an appropriate level of expertise to confirm that it is of an acceptable scientific standard.

## References

[1] : Revealing hidden depression in older people: a qualitative study within a randomised controlled trial. *BMC Family Practice* .2015;16(1) : 10.1186/s12875-015-0362-2 10.1186/s12875-015-0362-2 PMC461777726481581

# Reviewer report: Reviewer response for version 1

**DOI:** 10.21956/wellcomeopenres.27482.r142954

**Published:** 2026-02-20

**Authors:** Talitha Best

**Affiliations:** 1 School of Health, Medical and Applied Science, Central Queensland University, Brisbane, Queensland, Australia

Dear editorial team and authors, 

 Thank you for the opportunity to review this paper and the novel analysis in the pre-post war birth cohort of Scottish adults and the reported association between dietary intake and risk of anxiety and depression.

 Overall the approach to analysis is detailed, sound, well documented and overall clearly considered within the broader GLAD task force protocols and methodology described.

 Review comments for consideration are about the robustness of interpretation and rationale in the analysis.

 Namely:

- The small effect sizes and the absence of robust findings (none of the associations survived correction for multiple comparisons) has been considered well statistically. Though conceptually for interpretation, the absence of reference to the cohort themselves seems to underplay the potential relationship between the low prevalence rates of depression and anxiety and dietary habits formed in a generation that experienced significant changes in food and food tables. Might this be considered within this discussion?
- Similarly, the authors, whilst acknowledging the overall Scottish older-adult population dietary data, the lack of consideration of socioeconomic position and smoking behaviours in the adjustment does appear to limit interpretation. Could some context to this be provided?
- Further, the context of pre and post war food tables where the composition of 1930s – 1950’s foods were very different from how they are today (e.g. meat products) etc and type of dairy – how might using GBD 2019 tables for this cohort influence the context for the overall interpretation of this study’s findings?
- The low prevalence rates of depression and anxiety, yes may be due to small sample size but the study is well powered for the analysis conducted - What do the authors suppose may also be going on with the low prevalence rates of depression and anxiety? The comment that the sensitivity of the HADS may be contributing seems rather banal, given the measure has been used extensively in multiple cohorts and aged populations. Is there perhaps other contextual insights that might considered here for the cohort?Could the quality of foods and intake be a factor potentially? Please consider including some additional detail to consideration of measures and the context of their use.

- The interpretation about the depression and anxiety and poor dietary quality ( in general) in older Scottish adults seems to lack a clear relationship/reference/rationale in the analysis regarding consideration of other health comorbidities? For example ( not specified), Type 2 diabetes ( or CVD etc) and the relationship with dietary intake and mental health – could the rationale for why not included/considered  be included somewhere and describe what limitation this may place on interpretation and generalisability?
- Finally, the focus of the study is on individual components vs whole of diet patterns - how might this play a role in the interpretation of the findings and in what way might the authors provide a more in depth context for current findings?

Is the work clearly and accurately presented and does it cite the current literature?

Yes

If applicable, is the statistical analysis and its interpretation appropriate?

Yes

Are all the source data underlying the results available to ensure full reproducibility?

Yes

Is the study design appropriate and is the work technically sound?

Yes

Are the conclusions drawn adequately supported by the results?

Yes

Are sufficient details of methods and analysis provided to allow replication by others?

Yes

Reviewer Expertise:

Clinical psychology, behavioral and nutritional neuroscience, health behaviours, multiple methods/mixed methods research within interdisciplinary contexts

I confirm that I have read this submission and believe that I have an appropriate level of expertise to confirm that it is of an acceptable scientific standard.

# Response

**Published:** 2026-09-17

**Authors:** Janie Corley

**Affiliations:** Psychology, The University of Edinburgh Psychology Department, Edinburgh, Scotland, UK

**Response to reviewer** **Reviewer 1** *Thank you for the opportunity to review this paper and the novel analysis in the pre-post war birth cohort of Scottish adults and the reported association between dietary intake and risk of anxiety and depression. Overall the approach to analysis is detailed, sound, well documented and overall clearly considered within the broader GLAD task force protocols and methodology described. Review comments for consideration are about the robustness of interpretation and rationale in the analysis.*

**Response**: We thank the reviewer for their careful consideration of the manuscript, their positive feedback, and their helpful suggestions for improvement. We respond to each comment below, with the corresponding changes to the manuscript indicated in bold type after each response. All revisions are also shown using tracked changes in the revised manuscript.  

1. *The small effect sizes and the absence of robust findings (none of the associations survived correction for multiple comparisons) has been considered well statistically. Though conceptually for interpretation, the absence of reference to the cohort themselves seems to underplay the potential relationship between the low prevalence rates of depression and anxiety and dietary habits formed in a generation that experienced significant changes in food and food tables. Might this be considered within this discussion?*

**Response**: We thank the reviewer for this helpful observation and agree that the distinctive historical context of the LBC1936 warrants greater consideration when interpreting the findings. Participants were born in 1936 and therefore the generational experience of childhood and adolescence during the Second World War and post-war period, when food availability and prevailing dietary practices differed substantially from those experienced by later generations. Although dietary intake in the present study was assessed much later, at approximately age 70, these shared formative experiences may have influenced food preferences and dietary behaviours that persisted into later adulthood. We have therefore expanded the Discussion to acknowledge the potential importance of this birth-cohort context when interpreting the dietary profiles observed in the study. As earlier-life dietary intake was not examined in the present analysis, we have been careful not to infer a direct relationship between historical dietary exposures and the prevalence of anxiety or depression observed in this study. We also agree that the relatively low prevalence of depression in this cohort warrants broader consideration. We have therefore revised the Discussion to acknowledge several potentially relevant cohort-related factors, including healthy-participant and survival effects, as well as possible generational differences in the recognition or reporting of psychological symptoms, rather than focusing primarily on potential measurement-related explanations.

**Manuscript changes: “** *Furthermore, the relatively low prevalence of depression in the sample (4%)—substantially lower than the national average of around 9% for 65–74-year-olds in Scotland (Scottish Government, 2023)— **may have constrained the ability to detect associations. This relatively low prevalence may also reflect characteristics of the cohort itself, including healthy-participant and survival effects, as well as potential generational differences in the recognition or reporting of psychological symptoms. Study participants were born in 1936 and experienced childhood and adolescence during the Second World War and post-war period, when food availability and dietary practices differed substantially from those experienced by later generations. Although diet was assessed at approximately age 70 in the present study, these shared formative experiences may have contributed to dietary preferences and behaviours persisting into later life and provide important context for interpreting the dietary profiles and mental health outcomes observed in this cohort. However, as earlier-life dietary intake was not examined here, any relationship between these historical dietary exposures and later-life mental health remains speculative.”***  

1. *Similarly, the authors, whilst acknowledging the overall Scottish older-adult population dietary data, the lack of consideration of socioeconomic position and smoking behaviours in the adjustment does appear to limit interpretation. Could some context to this be provided?*

**Response**: We thank the reviewer for raising this important point. The primary covariate set of age, sex, and years of education was specified in accordance with the harmonised GLAD Taskforce analysis protocol to facilitate consistency across contributing cohorts. We agree that smoking and broader measures of socioeconomic position may be associated with both dietary intake and mental health and could therefore contribute to residual confounding. However, as none of the observed associations survived correction for multiple comparisons, we did not undertake further post hoc adjustment of the nominally significant associations with additional cohort-specific covariates, as we felt that this could place undue emphasis on findings for which the overall evidence was not robust. We have instead clarified the rationale for the covariate selection and expanded the Discussion to acknowledge the potential for residual confounding by smoking and aspects of socioeconomic position not captured by years of education, and the consequent limitation this places on interpretation. In addition, we have revised our interpretation of the association between sugar-sweetened beverage intake and depressive symptoms, removing the suggestion that the finding represented a potentially independent association and instead stating more precisely that the association was consistent following adjustment for age, sex, and years of education.

**Manuscript changes**: *“The absence of robust findings may reflect true null effects, weak effects that require greater statistical power than afforded in this setting, or methodological constraints, including the cross-sectional design and possible residual confounding.* ***Factors not included in the models, such as smoking, aspects of socioeconomic position not captured by years of education, and physical health comorbidities, may contribute to residual confounding…”*** ***“Nonetheless, the association was consistent following adjustment for age, sex, and years of education…”***  

1. *Further, the context of pre and post war food tables where the composition of 1930s – 1950’s foods were very different from how they are today (e.g. meat products) etc and type of dairy – how might using GBD 2019 tables for this cohort influence the context for the overall interpretation of this study’s findings?*

**Response**: We thank the reviewer for this point. We would like to clarify that dietary intake in the present analysis was not assessed during the participants’ childhood or the pre-/post-war period. The dietary exposures were derived from the FFQ completed at approximately age 70, during the LBC1936 baseline assessment in 2004–2007, which assessed habitual dietary intake over the preceding 2–3 months. Therefore, historical differences in the composition of foods consumed during the 1930s–1950s would not directly affect the dietary exposures examined in the present analysis. The GBD dietary definitions were applied to these later-life dietary data in accordance with the harmonised GLAD Taskforce protocol, enabling dietary exposures to be defined consistently across contributing cohorts. We nevertheless acknowledge that these broad dietary categories (e.g., milk, red meat and processed meat) do not capture all variation in the composition or type of foods consumed within each category. We have clarified this distinction in the Discussion and acknowledge this as a limitation when interpreting associations with individual dietary components. **Manuscript changes**: **“ *Although the use of GBD-defined dietary categories facilitated harmonisation across cohorts, these broad categories may not capture variation in the composition or type of foods consumed within individual categories, which should be considered when interpreting associations with specific dietary exposures*.”**  

1. *The low prevalence rates of depression and anxiety, yes may be due to small sample size but the study is well powered for the analysis conducted - What do the authors suppose may also be going on with the low prevalence rates of depression and anxiety? The comment that the sensitivity of the HADS may be contributing seems rather banal, given the measure has been used extensively in multiple cohorts and aged populations. Is there perhaps other contextual insights that might considered here for the cohort? Could the quality of foods and intake be a factor potentially? Please consider including some additional detail to consideration of measures and the context of their use.*

**Response**: We thank the reviewer for this comment. There are three parts, which we address in turn:

1. “ *What do the authors suppose may also be going on with the low prevalence rates of depression and anxiety?”* As noted in response to the reviewer’s earlier point, we agree that the relatively low prevalence of depression in particular warrants greater contextual consideration. As outlined above, we have amended the Discussion to reflect that we think that healthy participation bias, community-dwelling status and generational differences in symptom recognition and reporting might contribute to the lower-than-average prevalence rates.
2. *“The comment that the sensitivity of the HADS may be contributing seems rather banal, given the measure has been used extensively in multiple cohorts and aged populations”.  *We agree that the HADS has been extensively used and validated in older populations. However, since the GLAD analytic protocol reduces each score to a dichotomous split at the cut-off of ≥8, this results in reduced phenotypic variation in a cohort with a distribution already shifted towards the healthy end. That is, dichotomisation necessarily reduces information relative to use of the full continuous symptom scores and, particularly given the small number of participants meeting the threshold for depression, may have limited our ability to detect associations. We have now updated the text to clarify that we are not referring to the intrinsic properties of the HADS itself, but the implications of its use in the analytic and present cohort context.

**Manuscript changes**: from “… *and reliance on a brief screening tool (HADS) may have constrained the ability to detect associations.” To **“…and the use of dichotomised HADS outcomes may have limited the ability to detect associations, particularly given the small number of participants meeting the threshold for depressive symptoms.”***

1. “ *Is there perhaps other contextual insights that might considered here for the cohort? Could the quality of foods and intake be a factor potentially?*” We considered the reviewer’s suggestion that dietary quality might provide additional context. However, given the cross-sectional design, the present analyses cannot determine whether dietary quality contributed to the relatively low prevalence of depressive symptoms in this cohort. Moreover, dietary intake in the cohort was broadly suboptimal when evaluated against the GBD-defined thresholds. We have therefore avoided attributing the relatively low prevalence to dietary quality. The corresponding manuscript changes are provided in response to Comment 1 above.

 

1. *The interpretation about the depression and anxiety and poor dietary quality (in general) in older Scottish adults seems to lack a clear relationship/reference/rationale in the analysis regarding consideration of other health comorbidities? For example (not specified), Type 2 diabetes (or CVD etc) and the relationship with dietary intake and mental health – could the rationale for why not included/considered be included somewhere and describe what limitation this may place on interpretation and generalisability?*

**Response**: We thank the reviewer for this point. The primary covariate set of age, sex, and years of education was specified in accordance with the harmonised GLAD Taskforce analysis protocol to facilitate consistency across contributing cohorts. We agree that physical health comorbidities, including cardiometabolic conditions such as type 2 diabetes and cardiovascular disease, may be associated with both dietary intake and mental health and are therefore relevant to the interpretation of the findings. Although additional cohort-specific adjustment was possible, none of the observed diet–mental health associations survived correction for multiple comparisons. We therefore did not undertake further post hoc adjustment for individual health conditions of associations that were nominally significant but did not survive correction for multiple comparisons, as we considered that this could place undue emphasis on findings for which the overall evidence was not robust. In addition, the role of physical comorbidities is potentially complex, as some conditions may represent confounders, while others may lie on pathways linking long-term dietary exposures with mental health. We have revised the Discussion to acknowledge that physical health comorbidities were not included in the harmonised models and that this limits our ability to determine the extent to which the observed associations are independent of participants’ broader health status. This should therefore be considered when interpreting the findings and their generalisability.

**Manuscript changes**: *“The absence of robust findings may reflect true null effects, weak effects that require greater statistical power than afforded in this setting, or methodological constraints, including the cross-sectional design, possible residual confounding. Factors not included in the models, such as smoking, aspects of socioeconomic position not captured by years of education,* ***and physical health comorbidities**, may contribute to residual confounding, alongside measurement limitations of food frequency questionnaires and self-reported psychological constructs. **The role of physical health comorbidities is potentially complex as some conditions may act as confounders while others may lie on pathways linking long-term dietary exposures with mental health** **.”***  

1. *Finally, the focus of the study is on individual components vs whole of diet patterns - how might this play a role in the interpretation of the findings and in what way might the authors provide a more in depth context for current findings?*

**Response**: We thank the reviewer for raising this point and agree that the focus on individual dietary components is important when interpreting the findings. The present analyses examined individual GBD-defined dietary exposures in accordance with the harmonised GLAD Taskforce analysis protocol, rather than whole-diet patterns or dietary quality scores. This approach facilitates comparison of specific dietary exposures across contributing cohorts but does not capture the complexity of overall dietary patterns or the interrelationships between foods consumed within an individual’s diet. We have therefore expanded the Discussion to acknowledge that the nominal associations observed for individual dietary components may partly reflect broader dietary patterns or correlated dietary and lifestyle behaviours, rather than effects attributable to individual foods or nutrients in isolation. We also note that whole-diet approaches may capture cumulative or interactive relationships between dietary components and mental health that cannot be identified using the present component-based approach. This should be considered when interpreting the current findings. **Manuscript changes**: **“ *The present analysis focused on individual GBD-defined dietary components rather than whole-diet patterns. While this approach facilitates cross-cohort comparison of specific dietary exposures, foods and nutrients are not consumed in isolation, and individual components may also act as markers of broader dietary patterns. The nominal associations observed here may therefore partly reflect correlated dietary or lifestyle behaviours rather than relationships attributable to individual dietary components themselves. Whole-diet approaches may additionally capture cumulative or interactive relationships between dietary components and mental health that cannot be identified using the present component-based approach*.”** We have also revised the Conclusion to provide a more cautious interpretation of the findings, given that the observed associations were modest and did not survive correction for multiple comparisons. **Manuscript changes**: **“ *These findings warrant further investigation, but larger, longitudinal, and harmonised studies are needed to establish the direction of associations and strengthen causal inference*.”**
